# Mid-Term Outcomes of Aortic Valve Repair Without Aortic Root Replacement: A Single-Center Experience

**DOI:** 10.7759/cureus.63068

**Published:** 2024-06-24

**Authors:** Kenichi Kato, Naritomo Nishioka, Mika Yamamoto, Keita Sasaki, Ryo Matsumoto, Takahiko Masuda, Ryushi Maruyama, Yoshihiko Kurimoto, Shuichi Naraoka

**Affiliations:** 1 Department of Cardiovascular Surgery, Teine Keijinkai Hospital, Sapporo, JPN

**Keywords:** lifetime management strategy, valve durability, reoperation rate, aortic regurgitation, aortic valve repair

## Abstract

Background

Aortic valve (AV) repair is a challenging procedure due to its complexity, lower reproducibility, and steep learning curve. To examine its durability and validity, we investigated mid-term outcomes following AV repair without aortic root replacement.

Methods

Between March 2007 and May 2018, we retrospectively identified 14 patients who underwent AV repair without aortic root replacement at our institution. We investigated their baseline characteristics and postoperative outcomes, including the reoperation rate due to aortic regurgitation (AR) recurrence. Furthermore, we divided them into two groups: those who required reoperation due to AR recurrence (Group R) and those who did not require reoperation (Group F), and statistically compared them.

Results

The median age was 52.5 years (IQR: 42.0-60.8), with 11 male patients (78.6%). Eight patients (57.1%) had a bicuspid AV. Five cases (35.7%) underwent reoperation due to AR recurrence during a median follow-up period of 5.5 years. There were no significant differences in baseline characteristics between Group R (n=5, 35.7%) and Group F (n=9, 64.3%), including AR etiology, AV repair procedure, and intraoperative AR grade after the final declamp. All cases in Group R had at least mild to moderate AR on the echocardiogram before discharge. Regarding the AR grade before discharge, Group R had a significantly higher grade than Group F (p = 0.013).

Conclusions

The indication for AV repair for AR might need to be reassessed due to the considerable mid-term reoperation rate. Cases of AV repair with more than mild AR at discharge should be carefully monitored, as they are likely to require future reoperation for AR.

## Introduction

Aortic valve (AV) repair is a treatment option for aortic regurgitation (AR) in cases with no or mild cusp degeneration. This procedure employs various techniques, such as annuloplasty, repair with pericardium, and cusp plication, each tailored to the AR etiology in individual cases [1]. AV repair is particularly appealing for younger AR patients who would otherwise require a mechanical valve for aortic valve replacement (AVR), as it allows them to discontinue anticoagulant therapy after surgery. Despite these advantages, AV repair poses challenges, including its complex nature, lower reproducibility, and steep learning curve [2]. Studies have also indicated an increased likelihood of postoperative AR in patients undergoing AV repair without aortic root replacement [3]. At our institution, we have experienced several reoperations for AR recurrence after AV repair, even though the procedures were performed by surgeons with sufficient AV repair experience. Based on this, we generally do not perform AV repair today. To examine the durability and validity of this approach, we reviewed cases of AV repair without aortic root replacement performed at our hospital.

## Materials and methods

**Figure 1:**
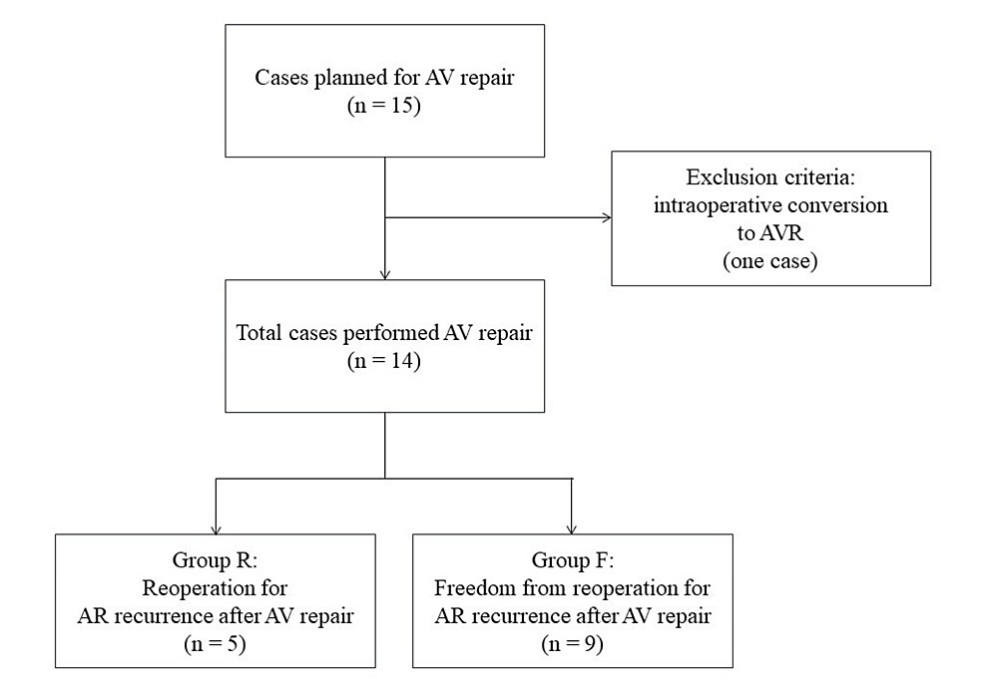
Flow chart of study population selection. Between March 2007 and October 2023, 15 cases were planned for AV repair without aortic root replacement at our hospital (Teine Keijinkai Hospital). Intraoperative conversion to AVR was chosen for one case, leaving 14 cases included in the analysis. These were divided into two groups: one group included patients who underwent reoperation due to AR recurrence after AV repair (Group R, n = 5); the other group included patients who did not require reoperation (Group F, n = 9). AR: Aortic regurgitation; AV repair: Aortic valve repair.

The Institutional Review Board of Teine Keijinkai Hospital approved this study (No. 2-023421-00) and granted a waiver for patient consent due to the provision of an opt-out option. Between March 2007 and May 2018, we identified 15 cases scheduled for AV repair without aortic root replacement at our hospital. One case was excluded due to an intraoperative conversion to AVR, leaving 14 cases for analysis (Figure 1). We retrospectively collected data on patient characteristics, intraoperative data including AV repair procedures, and postoperative data such as AR grade before discharge and duration. Additionally, we divided the patients into two groups: those who underwent reoperation due to AR recurrence after AV repair (Group R) and those who did not require reoperation (Group F). The two groups were statistically compared in terms of patient characteristics and outcomes to investigate the reasons for reintervention after AV repair.

AR grade was classified as none, mild, moderate, or severe, according to transthoracic echocardiography guidelines [4], with trivial AR considered as none. AR etiology was categorized as per a previous study [5]. AV repair included a combination of annuloplasty, repair with pericardium, central cusp plication, and tricuspidization. Annuloplasty was performed using either a subvalvular circular approach with an expanded polytetrafluoroethylene (ePTFE) strip [6] or an external suture technique with CV-0 Gore-Tex suture (WL Gore & Associates Inc., Newark, DE, US) [7], based on surgeon preference. The ePTFE strip was made by cutting a 0.6 mm thick Gore-Tex patch (WL Gore & Associates Inc., Newark, DE, US) into 5-9 mm wide strips. Central cusp plication utilized multiple single sutures of 6-0 Prolene (Ethicon Inc., Raritan, NJ, USA). Repair with pericardium involved cusp augmentation, cusp replacement, and fenestration repair using glutaraldehyde-treated autologous pericardium. Commissuroplasty was performed with 4-0 Prolene (Ethicon Inc., Raritan, NJ, USA) or 5-0 Nespylen (Alfresa Pharma Corporation, Osaka, Japan) with pledget as described in a previous report [8]. Tricuspidization, suited for bicuspid aortic valve cases, was performed by suspending the tip of the major cusp’s raphe with CV-5 Gore-Tex suture (WL Gore & Associates Inc., Newark, DE, US) [9]. Each duration for Groups R and F was measured from the date of the first operation to the date of reoperation or the last follow-up.

Statistical analysis

Continuous data are presented as median (IQR), while categorical data are presented as numbers (percentages). Continuous variables between the two groups were compared using the Mann-Whitney U test. Independent and paired categorical variables were compared with Fisher’s exact test and the Wilcoxon signed-rank test. The freedom from reoperation rate was obtained using the Kaplan-Meier method. All statistical analyses were performed using SPSS 21 (IBM Corporation, Armonk, NY, USA), and a p-value <0.05 was considered statistically significant.

## Results

Preoperative and intraoperative baseline characteristics are shown in Table 1. The median age was 52.5 years, with 11 males (78.6%). All cases were elective operations. Regarding AR etiology, type II (cusp prolapse) was the most frequent (n = 11, 78.6%) [5]. Some cases had multiple AR etiologies: type Ic + II in 2 cases, type Id + II in 1 case, and type Id + III in 1 case. Eight cases (57.1%) had a bicuspid aortic valve, all of which were type I in the Sievers classification [10]. In terms of the AV repair procedure, all cases underwent annuloplasty, with 6 cases (42.9%) using ePTFE strips and the remaining eight cases undergoing external suture annuloplasty. Central cusp plication, repair with pericardium, commissuroplasty, and tricuspidization were performed in six cases (42.9%), seven cases (50.0%), three cases (21.4%), and five cases (35.7%), respectively. Aortic cross-clamping occurred twice or more in four cases (28.6%). AR after the final declamp was controlled to no more than mild in all cases on intraoperative transesophageal echocardiogram. Five patients (35.7%) required reoperation due to AR recurrence after AV repair, with all reoperations involving aortic valve replacement. These patients were classified as Group R (n=5, 35.7%) and the other patients as Group F (n=9, 64.3%). No significant differences in patient characteristics, including AR etiology, AV repair procedures, and AR grade after the final declamp, were observed between Groups R and F.

**Table 1:** Preoperative and intraoperative baseline characteristics. AR: Aortic regurgitation; AV repair: Aortic valve repair; ePTFE: Expanded polytetrafluoroethylene; -: Statistically incomparable.

Baseline characteristics	AV repair cases (n = 14)		Group R (n = 5 (35.7%))		Group F (n = 9 (64.3%))		P-value
Age (year)-median (IQR)	52.5	(42.0-60.8)	58.0	(47.0-58.0)	42.0	(41-65)	0.797
Male-n (%)	11	(78.6)	4	(80.0)	7	(77.8)	0.725
Preoperative AR-n (%)							0.158
Moderate	5	(35.7)	1	(20.0)	4	(44.4)	
Moderate-Severe	3	(21.4)	0	(0)	3	(33.3)	
Severe	6	(42.9)	4	(80.0)	2	(22.2)	
AR etiology-n (%)							
Type Ic	2	(14.3)	1	(20.0)	1	(11.1)	0.604
Type Id	3	(21.4)	1	(20.0)	2	(22.2)	0.725
Type II	11	(78.6)	4	(80.0)	7	(77.8)	0.725
Type III	2	(14.3)	1	(20.0)	1	(11.1)	0.604
Bicuspid valve-n (%)	8	(57.1)	3	(60.0)	5	(55.6)	0.657
Annulus diameter (mm)-median (IQR)	26.1	(23.5-27.0)	26.8	(26.2-27.0)	25.9	(23.0-26.7)	0.364
Ejection fraction (%)-median (IQR)	58.7	(48.7-64.3)	56.7	(54.3-63.0)	60.7	(47.7-64.7)	1
Left ventricle diameter (mm)-median (IQR)							
End systolic diameter	46.7	(42.2-47.9)	46.4	(45.1-49.9)	46.9	(40.7-47.1)	1
End diastolic diameter	64.0	(58.4-67.7)	64.5	(63.5-70.9)	60.0	(58.1-66.6)	0.606
Procedure time (min)-median (IQR)							
Operation time	397	(325-466)	410	(341-521)	395	(310-423)	0.364
Cardiopulmonary bypass time	223	(142-289)	252	(172-336)	193	(138-268)	0.298
Aorta cross-clamp time	149	(96-215)	163	(135-242)	146	(94-183)	0.606
AV repair procedure-n (%)							
Annuloplasty	14	(100)	5	(100)	9	(100)	-
ePTFE strip	6	(42.9)	2	(40.0)	4	(44.4)	0.657
External suture	8	(57.1)	3	(60.0)	5	(55.6)	0.657
Central cusp plication	6	(42.9)	2	(40.0)	4	(44.4)	0.657
Commissuroplasty	3	(21.4)	2	(40.0)	1	(11.1)	0.275
Repair with pericardium	7	(50.0)	3	(60.0)	4	(44.4)	0.500
Tricuspidization	5	(35.7)	2	(40.0)	3	(33.3)	0.622
Other procedures-n (%)							
Mitral valve plasty	4	(28.6)	2	(40.0)	2	(22.2)	0.455
Cox-Maze IV procedure	3	(21.4)	1	(20)	2	(22.2)	0.725
Tricuspid valve annuloplasty	1	(7.1)	0	(0)	1	(11.1)	0.643
Aorta cross-clamp times-n (%)							0.685
Once	10	(71.4)	3	(60.0)	7	(77.8)	
Twice	3	(21.4)	2	(40.0)	1	(11.1)	
3 times	1	(7.1)	0	(0)	1	(11.1)	
Intraoperative AR after final declamp-n (%)							0.275
None	11	(78.6)	3	(60.0)	8	(88.9)	
Mild	3	(21.4)	2	(40.0)	1	(11.1)	

**Figure 2:**
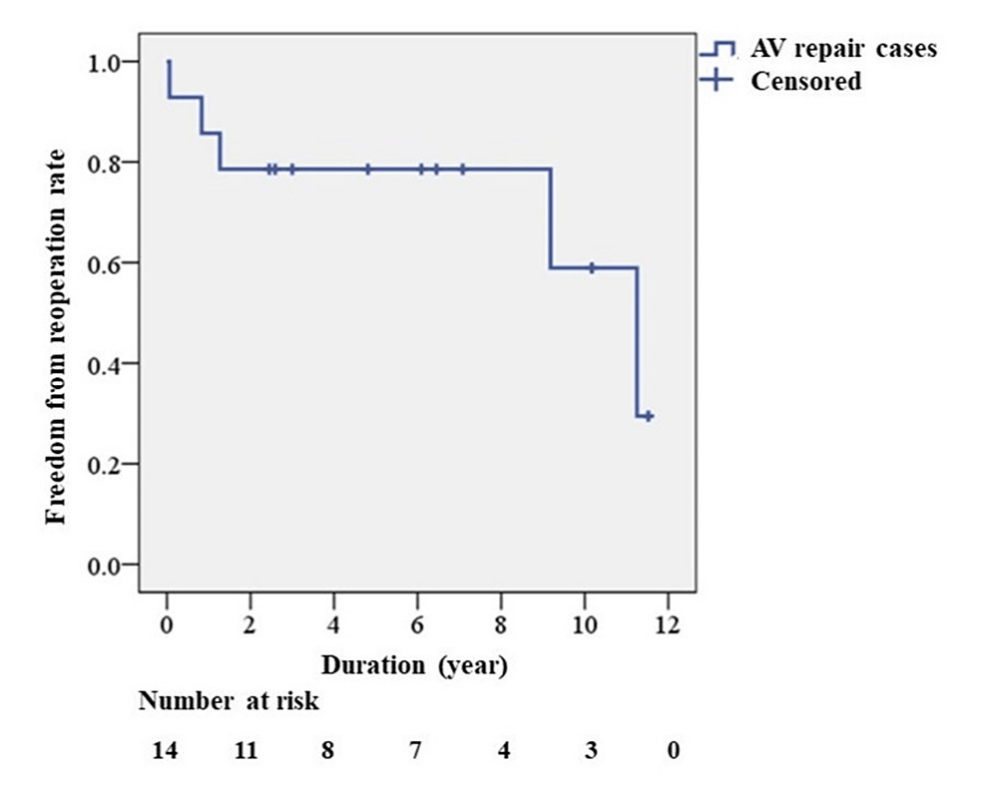
Kaplan-Meier curve of freedom from reoperation. AV repair: Aortic valve repair.

Postoperative outcomes are shown in Table 2. All cases in Group R had no less than mild to moderate AR at transthoracic echocardiogram before discharge, and their AR grade before discharge was significantly higher than that of Group F (p = 0.013). Eccentric AR before discharge, left ventricular ejection fraction, and diameter did not significantly differ between the two groups. The overall median follow-up period was 5.5 years (range 2.5-8.7), and the freedom from reoperation rate is shown in Figure 2. The intraoperative AR grade after the final declamp was significantly lower than the AR grade before discharge (p = 0.007, Table 3).

**Table 2:** Postoperative outcomes. †AV repair date ~ reoperation date, ‡AV repair date ~ last follow-up date *Statistically significant difference (p<0.05, Fisher's exact test). AR: Aortic regurgitation; AV repair: Aortic valve repair; -: Statistically incomparable.

Postoperative outcomes	AV repair cases (n = 14)		Group R (n = 5 (35.7%))		Group F (n = 9 (64.3%))		P-value
AR before discharge - n (%)							0.013*
None	4	(28.6)	0	(0)	4	(44.4)	
Mild	4	(28.6)	0	(0)	4	(44.4)	
Mild-Moderate	5	(35.7)	4	(80.0)	1	(11.1)	
Moderate-Severe	1	(7.1)	1	(20.0)	0	(0)	
Eccentric AR jet before discharge-n (%)	3	(21.4)	1	(20.0)	2	(22.2)	0.725
Ejection fraction (%)-median (IQR)	53.6	(44.4-62.2)	43.0	(41.5-55.4)	56.8	(50.7-64.4)	0.190
Left ventricle diameter (mm)-median (IQR)							
End systolic diameter	38.1	(31.5-41.9)	47.9	(43.2-52.6)	36.4	(34.0-38.8)	0.286
End diastolic diameter	53.2	(48.2-56.2)	56.3	(55.8-62.4)	50.2	(47.5-54.6)	0.112
Duration (year)-median (IQR)	5.5	(2.5-8.7)	1.3	(0.8-9.2)^†^	6.1	(3.0-7.1)^‡^	-

**Table 3:** Comparison of AR grade after the final intraoperative declamp and before discharge. *Statistically significant difference (p<0.05, Wilcoxon signed-rank test). AR: Aortic regurgitation; AV repair: Aortic valve repair.

AV repair cases (n=14)	Intraoperative AR after final declamp		AR before discharge		P-value
AR grade-n (%)					0.007*
None	11	(78.6)	4	(28.6)	
Mild	3	(21.4)	4	(28.6)	
Mild-Moderate	0	(0)	5	(35.7)	
Moderate-Severe	0	(0)	1	(7.1)	

All cases of Group R are summarized in Table 4. Two cases had ePTFE annuloplasty strip and tricuspidization suture detachment, leading to reoperation approximately 10 years after AV repair. Another two cases, who underwent reoperation approximately one year after AV repair, had prolapse of pericardial patch-augmented cusps. The remaining case had central cusp plication suture detachment, resulting in reoperation 20 days after AV repair with continued hospitalization.

**Table 4:** Details of each case in Group R. †AV repair date ~ reoperation date AR: Aortic regurgitation; AV repair: Aortic valve repair; ePTFE: Expanded polytetrafluoroethylene; Maze: Cox-Maze IV procedure; MVP: Mitral valve plasty.

Group R cases	No. 1	No. 2	No. 3	No. 4	No. 5
Age (year)	47	45	58	60	58
Sex	Male	Male	Male	Female	Male
Bicuspid valve (Sievers type)	Yes (Type I, L/R)	Yes (Type I, L/R)	Yes (Type I, L/R)	No	No
Preoperative AR	Severe	Severe	Moderate	Severe	Severe
Annulus diameter (mm)	32.0	27.0	26.2	21.1	26.8
AR etiology	Type Ic + II	Type II	Type Id + III	Type II	Type II
AV repair procedure					
Annuloplasty	ePTFE strip	ePTFE strip	External suture	External suture	External suture
Central cusp plication	No	No	Yes	No	Yes
Commissuloplasty	No	No	Yes	Yes	No
Repair with pericardium	No	Yes	Yes	Yes	No
Tricuspidization	Yes	Yes	No	No	No
Other procedures	No	MVP	MVP + Maze	No	No
Aorta cross-clamp times	Once	Once	Once	Twice	Twice
Intraoperative AR after final declamp	Trivial	Trivial	Mild	Mild	Trivial
AR before discharge	Mild-Moderate	Mild-Moderate	Mild-Moderate	Mild-Moderate	Moderate-Severe
AR before reoperation	Severe	Severe	Severe	Severe	Moderate-Severe
Etiology of AR recurrence	ePTFE strip and tricuspidization suture detachment	ePTFE strip and tricuspidization suture detachment	Augmented cusp prolapse	Augmented cusp prolapse and shortening	Central plication suture detachment
Duration^†^	9.2 years	11.2 years	1.3 years	10 months	20 days

## Discussion

The major findings of the present study are as follows: Five cases (35.7%) underwent reoperation due to AR recurrence during a median follow-up period of 5.5 years. In cases requiring reoperation, the AR grade before discharge was no less than mild to moderate, although the intraoperative AR grade was no more than mild. Identifying factors associated with reoperation proved difficult.

A previous study involving 331 AV repair cases without aortic root replacement reported late reoperation rates at 5, 10, and 15 years as 10%, 21%, and 28%, respectively [11]. The results of our study appear inferior compared to these previous results from an experienced high-volume center, potentially attributable to differences in institutional experience. Conversely, the reoperation rates at 10, 15, and 20 years after AVR with a bioprosthetic valve (Carpentier-Edwards Perimount pericardial bioprosthesis, Edwards Lifesciences, Irvine, CA) have been reported as 6.8%, 18.5%, and 48.5%, respectively [12]. These percentages for reoperation at 10 and 15 years after the initial operation are lower than those of AV repair. Moreover, newer bioprosthetic valves with anti-calcification treatments, such as capping of residual aldehyde groups and glycerin treatment, are now available, including the INSPIRIS RESILIA Aortic Valve (Edwards Lifesciences, Irvine, CA). Although only short to mid-term results have been reported thus far [13], they are expected to have better durability than previous bioprosthetic valves. Additionally, transcatheter aortic valve implantation (TAVI) in surgical aortic valve (TAV-in-SAV) has become an option for treating surgical valve deterioration. The concept of a 'lifetime management strategy' is emerging [14], emphasizing a treatment plan from the first intervention that considers the possibility and methods for future re-intervention. Given that TAVI is not currently indicated for AR, including recurrence after AV repair, and bioprosthetic aortic valves are recommended for patients over 50 years old based on the 2020 American College of Cardiology/American Heart Association guideline [15], AVR with bioprosthetic valves may be a more reasonable option for such cases, especially if an adequately-sized valve for future TAV-in-SAV can be implanted. The indication for AV repair appears limited; however, AV repair, which preserves the native cusps, may ideally allow a lifetime without reoperation, unlike bioprosthetic valves, which inevitably deteriorate. It may be a useful option for patients younger than 50 years with mild valve degeneration, sufficient cusp height, and good prospects for plasty. This may be particularly beneficial for young women aiming to conceive, as they can avoid taking warfarin. In any case, AV repair should be performed with accurate evaluation of the valve components such as annuls, commissure, leaflet, sinus, and sinotubular junction as a unit at institutes with substantial AV repair experience and by surgeons with ample experience.

Several risk factors for reoperation after AV repair have been reported, including cusp suture repair, larger left ventricle end-diastolic volume, commissuroplasty, and younger age [3]. However, in the present study, no significant differences in preoperative and intraoperative baseline characteristics, including AR etiology, echocardiogram data, and AV repair procedure, were observed between Groups R and F. This lack of difference might be due to the limited number of cases in this study.

The postoperative AR grade before discharge in Group R was more than mild in every case and significantly higher than that of Group F. A previous study reported that more than mild AR at discharge after AV repair was a predictor of late reoperation [11]. AV repair cases with an AR grade of more than mild at discharge require careful follow-up. However, the intraoperative AR grade after the final declamp was significantly lower than the postoperative grade before discharge. In contrast, no significant difference was observed in intraoperative AR between the two groups. The reason why the intraoperative AR grade was underestimated might be due to comparative hypotension under general anesthesia and decreased cardiac function immediately after declamp. It is difficult to make an intraoperative decision to convert to AVR based on the AR grade, with almost no difference between the two groups.

Among the cases in Group R, ePTFE annuloplasty strip and tricuspidization suture detachment were observed in two patients. Specifically, two (33.3%) out of six cases with ePTFE strip annuloplasty and two (40.0%) out of five cases with tricuspidization underwent reoperation. Both underwent reoperation approximately 10 years after AV repair. It has been reported that the rate of freedom from reoperation two years after subvalvular circular annuloplasty with ePTFE strip and leaflet suspension was 88.9 ± 7.4% [16]. However, its long-term outcomes have not yet been reported. The mid- to long-term outcomes of tricuspidization are also unclear. The durability of ePTFE strip annuloplasty and tricuspidization with CV-5 Gore-Tex suture are questionable. A previous study reported that double ring annuloplasty (supravalvular ring annuloplasty at the sinotubular junction with subvalvular ring annuloplasty) is associated with better outcomes compared to single subvalvular annuloplasty [17]. Additional supravalvular annuloplasty may improve durability. Moreover, prolapse of the pericardial patch-augmented cusp approximately 1 year after AV repair was observed in two cases. It has been reported that the reoperation rate at one and six years after the Ozaki procedure, an aortic valve reconstruction procedure using autologous pericardium, was 0.5% and 3.2%, respectively [18]. Considering the durability of autologous pericardium, reoperation at one year after AV repair might be attributed to insufficient plasty rather than pericardial degeneration.

This study has several limitations. It is a retrospective cohort and a single-center study with a small sample size. The AV repair procedure varied depending on the surgeons' preferences. In addition, cusp height could not be investigated as only a few cases had cusp height measured on the preoperative echocardiogram. It has been reported that AR cases with a cusp geometric height of 16 mm or less in the tricuspid valve and 19 mm or less in the bicuspid aortic valve are mostly considered unsuitable for AV repair and opt for AVR [19]. As geometric height measurements were not performed in all cases, this might have contributed to AR recurrence in this study.

## Conclusions

The mid-term reoperation rate after AV repair appears to be considerable. The indication for AV repair in AR cases should be carefully considered. It should be kept in mind that intraoperative AR after declamp during AV repair tends to be underestimated compared to AR before discharge. AV repair cases with more than mild AR at discharge are likely to undergo reoperation for AR recurrence in the future and should be carefully monitored. However, this study failed to identify any preoperative or intraoperative factors that would predict postoperative AR recurrence. A larger-scale study will be needed to detect them.
